# Therapeutic Effect of Catgut Implantation at Acupoint in a Mouse Model of Hepatocellular Carcinoma by Suppressing Immune Escape

**DOI:** 10.1155/2022/5572869

**Published:** 2022-02-08

**Authors:** Shi-Hua Xu, Hao-Xuan Luo, Bi-Jun Huang, Ling Yu, Shao-Ju Luo, Hao Hu, Yan Li, Xiao-Tong Lin, Zhi-Rui Cao, Yuan-Jiang Deng, Shi-Jun Zhang

**Affiliations:** ^1^Department of Traditional Chinese Medicine, The First Affiliated Hospital, Sun Yat-Sen University, Guangzhou, Guangdong 510080, China; ^2^Department of Nephrology, Chongqing City Hospital of Traditional Chinese Medicine, Chongqing, Chongqing 400021, China; ^3^Department of Experimental Research, State Key Laboratory of Oncology in South China Collaborative Innovation Center for Cancer Medicine Sun Yat Sen University Cancer Center, Guangzhou, Guangdong 510060, China; ^4^Department of Pathology, The First Affiliated Hospital, Sun Yat-Sen University, Guangzhou, Guangdong 510080, China

## Abstract

**Background:**

The occurrence and development of hepatocellular carcinoma (HCC) are closely related to immune function, as is the capacity of hepatoma cells to escape. Immunosurveillance is a key mechanism. Catgut implantation at acupoint (CIAA) is a promising acupuncture improvement method that can regulate immunity and has been widely used in the clinical treatment of a variety of diseases. The aim of this study is to observe the therapeutic effect of CIAA on HCC and to investigate the potential mechanism of immune escape.

**Materials and Methods:**

A total of 40 mice were randomly divided into three groups: the HCC model group (*n* = 15), the CIAA treatment group (*n* = 15), and the control group (*n* = 10). HCC was chemically induced in 30 mice by the combination of DEN, carbon tetrachloride, and ethanol for 150 days. Among them, 15 were selected for CIAA treatment to ascertain the therapeutic effect. The mRNA expression levels of AFP, IL-10, PD-1, and CTLA-4 in three groups were examined by using RT-PCR. AFP and AKT expressions were measured by using western blotting. PD1, CTLA-4, IL-10, CD4+, and CD8+ protein expression levels were evaluated by using IHC. The mortality rate, body weight, and psychological conditions of three groups were also compared.

**Results:**

The mRNA and protein expression levels of AFP, PD-1, CTLA-4, and IL-10 were significantly downregulated in the CIAA-treated mice in comparison with HCC mice. IHC assay shows that CD4+ and CD8+ expression levels were notably upregulated after CIAA treatment. Western blotting assay shows that AKT pathway was deactivated in CIAA-treated mice. CIAA notably reduced the mortality rate and inhibited weight loss caused by HCC and improved the overall psychological condition of the mice.

**Conclusions:**

Taken together, our data corroborate the effective potency of CIAA in the treatment of HCC by and inhibiting immune escape and deactivating the AKT pathway.

## 1. Introduction

Hepatocellular carcinoma (HCC) occurs after years of liver disease and has become the most prevalent form of primary liver cancer on a global scale [1, 2]. To date, many treating strategies have been extensively used, such as hepatic resection, orthotopic liver transplantation, transarterial embolization, chemoembolization, and targeted agent therapy [3–5]. However, conventional chemotherapy drugs have high systemic side effects and poor local selectivity, and the survival rates of HCC patients remain poor [6]. Thus, the dismal prognosis for most HCC patients indicates a pressing need to develop novel and efficient therapeutic strategies against HCC.

Immune escape, also known as immune suppression or immune evasion, manifests the incapacity of the immune system for the removal of transformed or mutant cells and thus is a hallmark of carcinogenesis [7, 8]. An immune escape mechanism is implicated in the onset and progression of tumors and is characterized as a notable phenotype of malignancies. It mainly includes antigen deletion or antigen modulation; low expression of MHC-I molecules; abnormal costimulatory signals; expression or secretion of immunosuppressive factors; antiapoptotic effect; and tumor-induced immune cell apoptosis and suppression [9]. The immune escape of tumor cells is also closely related to the hypoxic/acidic immunosuppressive microenvironment.

As the main types of tumor-infiltrating lymphocytes (TIL), CD8 and CD4 T cells are deeply implicated in the cellular immune response. CD8 can bind with MHC class I antigen fragments, embed into tumor cells to form a tubular structure, and then release perforin into tumor cells to induce the lysis of tumor cells [10]. Moreover, CD4 + cells, as antigen-presenting cells, bind to MHC class II antigen fragments and participate in immune response. IL-10 is an important immunosuppressive factor released by tumor cells during the progression of malignancies [11].

PD-1 and CTLA-4 are known as the intrinsic checkpoint mediators that play key roles in the suppression of innate immune sensing. PD-1 and CTLA-4 can bind to ligands on tumor cells, thereby inhibiting the immune response mediated by T cells [12].

The expression patterns of PD-1 and CTLA-4 could be indicative of the detection and clearance of tumoral cells through the production of strong coinhibitory signals from T cells. CTLA-4 is expressed on the surface of activated T lymphocytes and plays an immunosuppressive role after binding with the B7 molecule on the surface of antigen-presenting cells (APCs) [13]. Both CTLA-4 and CD28, which share a high degree of homology, are expressed on the surface of T cells. However, CTLA-4 is a synergistic inhibitor to suppress the activation of T cells, whereas CD28 is a costimulatory factor which combines with B7 to promote the activation of T cells [14].

As an evolutionarily conserved serine protein kinase from the subfamily of protein kinase AGC subfamily, AKT plays a catalytic role via the phosphorylation of serine by mTOR Complex2 at Ser473(AKT1), Ser474(AKT2), and Ser472(AKT3) [15], thereby mediating a number of vital downstream effector molecules via phosphorylation cascade reaction to control several biological events in the cells, such as growth, proliferation, survival, glucose metabolism, and neovascularization. Nevertheless, the activities of these phosphatases can be frequently missed out or inactivated in human cancer, which is followed by the event of AKT hyperactivation [16]. Massive researches have confirmed the implication and aptitude of the PI3K/AKT pathway in preclinical experiments or trials, highlighting its druggable value for the anticancer treatment [17]. In the current study, we evaluate the abnormal p-AKT expression in the aberrant signaling cascades in HCC to evaluate the benefitting effect of CIAA treatment. Mitsiades et al. indicated that AKT and its upstream regulators are aberrantly regulated in several tumors and malignancies, and that the AKT pathway is a vital determinant of biologic aggressiveness of tumors [18]. These pieces of evidence suggest that AKT is a major potential target for novel anticancer therapies.

Acupuncture and moxibustion have a long history in the treatment of cancer to improving the life quality of patients. Its benign, bidirectional, and holistic treatment characteristics can resist external impairment by mobilizing the right qi of the human body itself. It has been reported that acupuncture increases the levels of CD3^+^ and CD4^+^ and the CD4^+^/CD8^+^ ratio in the peripheral blood of patients with malignant tumors, highlighting the therapeutic effect of acupuncture by enhancing the cellular immune function of the patients. Catgut implantation at acupoint (CIAA) is a method of injecting absorbable catgut into acupoints, an extension and important part of traditional acupuncture and moxibustion methods. Prior research found that CIAA therapy at Yingxiang (LI20) and Zusanli (ST36) acupoints effectively attenuated allergic symptoms and inflammatory parameters in the rat model of AR, highlighting the role of CIAA treatment as an alternative therapeutic in AR [19]. The experimental results show that CIAA is capable of improving the immune status of the body by increasing the activity of NK cells, enhancing the phagocytic function of macrophages, and increasing the level of T lymphocyte subsets [20].

Diethylnitrosamine (DEN) and carbon tetrachloride (CCl_4_) are well-known chemical agents which exhibit potent hepatocarcinogenic effects to induce HCC in an experimental mouse model, which is quite similar to the case of human HCC [21]. Accordingly, we established this model to investigate the antitumor effect of CIAA on DEN\CCl_4_\ethanol-induced HCC mice by detecting the expression levels of AFP, CD4^+^, CD8^+^, IL-10, PD-1, CTLA-4, and AKT to explore the possible underlying molecular mechanisms of the therapeutic effect of CIAA.

## 2. Materials and Methods

### 2.1. Reagents

Diethylnitrosamine (DEN) was purchased from Sigma (N0258, Sigma-Aldrich, USA). Carbon tetrachloride was purchased from Hao-Sen Chemical Co., Ltd. (Shanghai, China). Ethanol (55%) was purchased from Niu Lan Shan Co., Ltd. (Beijing, China). The TRI Reagent was purchased from Sigma (T9424; Sigma-Aldrich, USA). The Revert Aid First Strand cDNA Synthesis Kit was purchased from Thermo Fisher (K1621, Thermo Fisher; U.S.A). HieffTM qPCR SYBR® Green Master Mix was purchased from Yeasen Biotech Co., Ltd. (Cat. No. 11203, Shanghai, China). Anti-pan-AKT antibody (#ab8805, Abcam, Cambridge, U.K.), anti-p-AKT (ser473) antibody (#ab126433, Abcam, Cambridge, U.K.), Anti-AFP primary antibody (cat# ab46799, Abcam, Cambridge, U.K.), *β*-actin antibody (cat# ab8227, Abcam, Cambridge, U.K.), anti-IL-10 primary antibody (cat#ab189392, Abcam, Cambridge, U.K.), and CTLA-4 antibody (cat#ab134090, Abcam, Cambridge, U.K.) were purchased from Abcam (UK). CD4 (cat#25229), CD8*α* (cat#98941), and PD-1 (cat#84651) antibodies were purchased from Cell Signaling Technology (M.A. USA).

### 2.2. Animal Preparation

Male Balb/*c* mice (18–22 g, six weeks of age) were purchased from the Nanjing Biomedical Research Institute of Nanjing University (Certification No. SCXK (Su) 2015–0001) and were maintained under pathogen-free conditions in the Animal Experimental Center of Sun Yat-Sen University (Certification No. SYXK (Yue) 2015–0108). All mice were housed in a dry SPF environment between 24°C and 26°C with a 12 h light/dark cycle and were fed ad libitum. Before the experiment was carried out, all mice were adapted for 1 week. All procedures were monitored strictly by the Institutional Animal Care and Use Committee (IACUC), Sun Yat-Sen University according to the ‘3R' principles (replace, reduce, and refine) of humanistic care to experimental animals (Ethical Approval No. IACUC-2017-120).

Mice were randomly divided into three groups: the control group (*n* = 10), the HCC model group (*n* = 15), and the CIAA treatment group (*n* = 15). For the control group, we injected the same amount of saline and intragastrically administrated olive oil. For the HCC model group, the mice received an intraperitoneal injection of DEN at an initial dose of 95 mg/kg on the first day, followed by intragastric administration of CCl_4_/olive oil mix (1 : 4) at 5 ml/kg on the 4th day twice a week, a lower I/P dose of DEN (50 mg/kg) and intragastric administration of spirit at 50 ml/kg from the 3rd week, and finally 8 ml/kg of CCl_4_/olive oil mix and 7 ml/kg spirit from the 4th week onward.

For the CIAA treatment group, the mice received the same procedure as the mice in the HCC group, but in the last 60 days, they were treated with catgut embedding every 10 days for 6 times. In the end of the experiment, we evaluated the psychological conditions of the mice according to the standard elaborated in Table 1.

### 2.3. The CIAA Method

According to the acupoints described in previous study [22], the acupoints were also located on the basis of comparative anatomy and bone size division. We select on both sides “Zusanli” (ST36) and “Guan Yuan” (RN4) for catgut embedding at acupoints. First of all, we disinfected the acupoints using 75% alcohol, then cut the 4–0 catgut to a length of 2–3 mm, and rinsed with normal saline, inserted into the No. 7 buried wire needle, then penetrated into the acupoint at about 4 mm depth, pushed the catgut of appropriate length into the tissue according to the muscle abundance of the corresponding acupoint, then slowly pulled out the embedded wire needle after the catgut had been implanted tissue, and observed the acupoint again after drawing out. To ensure that the intestinal thread implantation is not taken out, press the needle hole for a moment after the needle is out.

### 2.4. Quantitative Real-Time Polymerase Chain Reaction

The RNA was extracted from liver tissue using TRIzol reagent, and cDNA was generated according to the manufacturer's instructions. The gene expression levels were measured by SYBR green-based three-step real-time PCR. The PCR primers used were as follows: mAFP, forward 5′-CTTCCCTCATCCTCCTGCTAC-3′ and reverse 5′-ACAAACTGGGTAAAGGTGATGG-3′; IL-10, forward 5′-GCTCTTACTGACT GGCATGAG-3′ and reverse 5′-CGCAGCTCTAGGAGCATGTG-3′; PD-1, forward 5′-ACCCTGGTCATTCACTTGGG-3′ and reverse 5′-CATTTGCTCCCTC TGACACTG-3′; CTLA-4, forward 5′-GCTTCCTAGATTACCCCTT CTGC-3′ and reverse 5′-CGGGCATGGTTCTGGATCA-3′; *β*-actin, forward 5′-GTGACG TTGACATCCGTAAAGA-3′ and reverse 5′-GCCGGACTCATCGTACTCC-3′. The PCR reaction was conducted at 95°C for 5 min, followed by 40 cycles of 95°C for 10 sec, 60°C for 20 sec, and 72°C for 20°s, ending with 15 sec at 95°C, 60°C for 60°s, and 95°C for 15 sec. The 2-ΔΔ^ct^ method was used to calculate the fold changes in gene expression levels.

### 2.5. Western Blotting

Protein was extracted from liver tissues using RIPA buffer with PMSF. Briefly, 200 mg of liver tissue of each sample was homogenized in the RIPA-PMSF mix (ratio of liver tissue and RIPA was 1 : 5, and that of liver tissue and PMSF was 20 : 1) on ice and centrifuged. The protein concentration of the homogenates was measured with the BCA method. Sample buffer was added at a ratio of 5 : 1, and the proteins were denatured at 95°C for 10 min. SDS-PAGE and membrane transfer were performed by standard protocols. The membranes were incubated overnight with the primary antibody, washed with TBST, incubated with the secondary antibody at 37°C for 1°h, washed again with TBST, and developed using ECL fluorescent substrate. Protein bands were visualized using X-ray photography, and the developed X-ray film was scanned by a high-resolution scanner. The grayscale values of the bands were measured and statistically analyzed by using Image *J* software.

### 2.6. Histopathology Analysis (HE)

Liver samples were fixed in 10% formalin immediately and embedded in paraffin. Representative histological sections (4 µm in thickness) were obtained and stained with hematoxylin and eosin for examination. The changes of liver pathology and the extent of liver tumors were observed and diagnosed under a microscope.

### 2.7. Immunohistochemistry (IHC)

IHC was performed with the suitably diluted antibodies at 37°C for 1h. After 5 2 min washes in PBS buffer, the slides were incubated with the secondary antibody at 37°C for 30 min. After another series of PBS washes, the sections were stained with DAB, counterstained with hematoxylin, dehydrated, and mounted for viewing. The presence of brownish granules in the cell membrane, cytoplasm, or nucleus was defined as positively stained cells.

### 2.8. Statistical Analysis

All the experimental data were analyzed using GraphPad Prism 8 and were reported in the form of mean ± SD based on triplicated assays. Categorical variables were tested using a chi-square test, whereas continuous variables were tested using ANOVA or two-tailed independent sample *t*-test. In the case of a nonnormal distribution, the Wilcoxon rank sum test was used, and the correction *t*-test was used when the variance was not homogeneous. The hypothesis test was set to *P* < 0.05, which was statistically significant.

## 3. Result

### 3.1. Effect of CIAA on Hepatocarcinogenesis in HCC Mice

**Figure 1:**
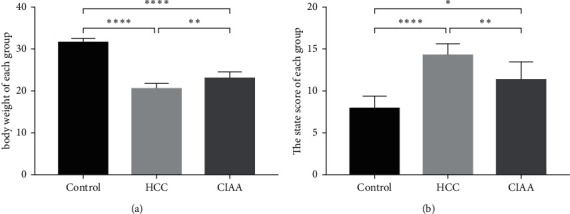
Effect of CIAA on hepatocarcinogenesis in HCC mice. (a) Comparison of body weight in each group of mice (control vs. HCC, *P* < 0.0001; HCC vs. CIAA, *P* < 0.005; control vs. CIAA, *P* < 0.0001); (b) Comparison of body condition scores of each group (control vs. HCC, *P* < 0.0001; HCC vs. CIAA, *P* < 0.01; control vs. CIAA, *P* < 0.05).

To investigate the antitumor effects of CIAA in HCC mice, CIAA was carried out on the 90th day after combined induction. The mice in the CIAA group were treated with CIAA every ten days for six times until the end of the experiment. The mortality, body weight, and state of each group were analyzed. Compared with the HCC group, CIAA significantly reduced the mortality (Table 2). We found that the body weight of the CIAA group was significantly higher than that of the HCC group (*P* < 0.005). Moreover, we scored the general state of the mice in each group (Table 1) and found that the body condition score of group CIAA was significantly better than that of group HCC (*P* < 0.05) (Figure 1).

### 3.2. Effect of CIAA on Hepatic Histopathology in HCC Mice

**Figure 2:**
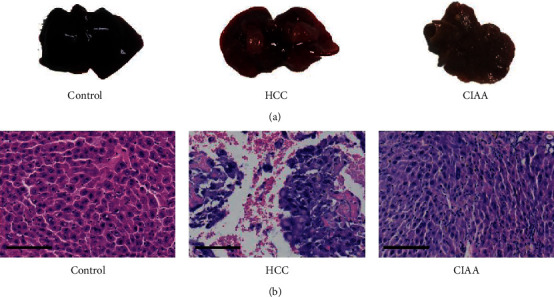
Effect of CIAA on hepatic histopathology in HCC mice. (a) The liver of each group observed with the naked eye; (b) HE staining in the liver tissue of mice in each group (scale bar = 100 *μ*m).

The morphological changes of liver tissues in each group were elaborated as follows: Observation with the naked eye shows the liver tissue of the control group was dark and smooth, whereas the large masses and rough surface can be seen in the liver tissue of HCC mice. The liver from the CIAA group appeared light in color, with numerous white nodules of different sizes appearing on the surface. The hepatic histopathology of various groups of mice was examined by H and E staining. Microscopically, the hepatic sections of the liver tissue from the control group revealed normal liver parenchyma, which is characterized by typical hepatic lobules and small uniform nuclei. However, in the HCC group, the liver cells were disordered, nuclear large, deeply stained, and irregular in shape. Mitotic image and basophilic change of cytoplasm were observed, which were consistent with the morphological characteristics of hepatocellular carcinoma. In the CIAA group, we observed the hepatic cord and sinuses of mice were bile embolism and cholestasis, and the nuclei were obviously enlarged, mostly round and binucleated hepatocytes. The hepatic pathocells infiltration and hydropic degeneration were gradually improved in comparison with the HCC group (Figure 2).

### 3.3. Effect of CIAA on the Expression of AFP and AKT in HCC Mice

**Figure 3:**
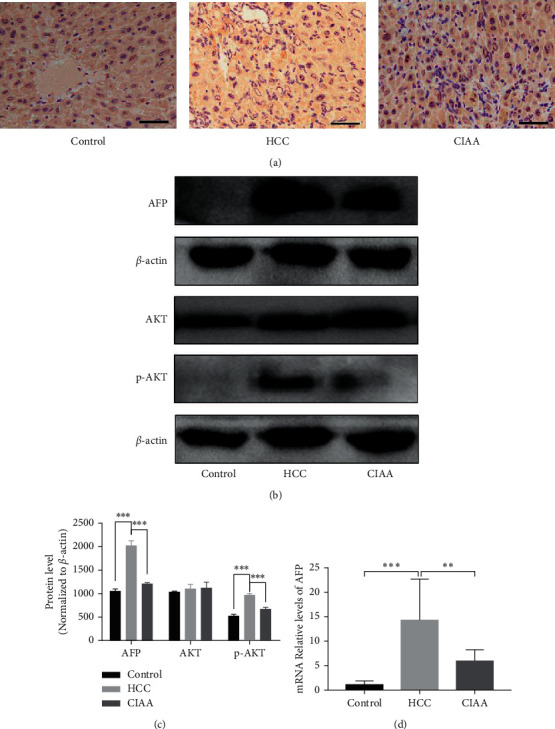
Effect of CIAA on the expression of AFP and AKT in HCC mice. (a) AFP protein expression and distribution in live tissue (scale bar = 100 *μ*m); (b) AFP and AKT pathway protein expression in the liver tissue; (c) protein level relative expression of the western blot (data were presented as the mean ± standard deviation. ^*∗∗∗*^*p* < 0.001 < 0.001, normalized to *β*-actin); (d) AFP mRNA expression in the liver tissue (control vs. HCC, *P* < 0.001; HCC vs. CIAA, *P* < 0.01; control vs. CIAA, *P* < 0.001).

AFP protein and mRNA expression was assessed using western blotting, IHC, and qPCR, respectively. The mRNA expression of AFP in the HCC group was significantly higher than that in the control group (*P* < 0.001), while the expression of AFP in the CIAA group was lower than that in the HCC group (*P* < 0.01). AFP was mainly distributed in the cytoplasm of HCC, and the expression of AFP in the HCC group was stronger than that in the control group. After the thread embedding, AFP expression in the CIAA group decreased to a certain extent. The expression of AKT and p-AKT was assessed by using western blotting. The protein expression of p-AKT in the HCC group was significantly higher than that in the CIAA group (Figure 3).

### 3.4. Effect of CIAA on Immune Status in HCC Mice

**Figure 4:**
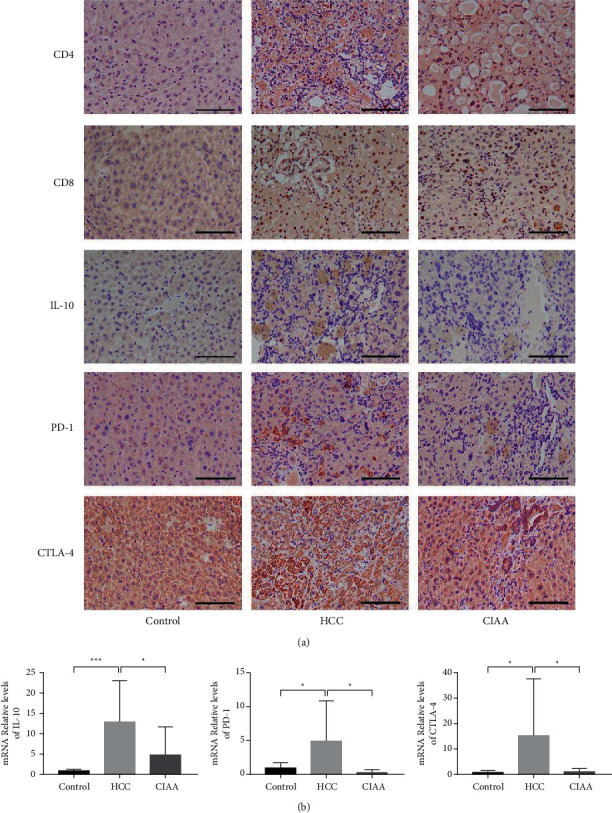
Effect of CIAA on immune status in HCC mice. (a) Immunohistochemical results of CD4, CD8, IL-10, PD-1, and CTLA-4 in liver tissue; (b) qPCR results of IL-10, PD-1, and CTLA-4 in liver tissues (mRNA of IL-10 : control vs. HCC, *P* < 0.0005; HCC vs. CIAA, *P* < 0.0005; control vs. CIAA, *P* > 0.05; mRNA of PD-1 : control vs. HCC, *P* < 0.05; HCC vs. CIAA, *P* < 0.05; control vs. CIAA, *P* < 0.01; mRNA of CTLA-4 : control vs. HCC, *P* < 0.05; HCC vs. CIAA, *P* < 0.05; control vs. CIAA, *P* > 0.05).

The protein expression levels of CD4^+^, CD8^+^, IL-10, PD-1, and CTLA-4 were assessed using IHC. The mRNA expression levels of IL-10, PD-1, and CTLA-4 were assessed using RT-PCR. The results showed that CD8^+^ was primarily expressed on the cell membrane of TILs. No CD8^+^ was found to be expressed in the liver tissue of the control group. The expression of CD8^+^ in TILs was observed in both the HCC group and the CIAA group. The expression of CD8^+^ in the CIAA group was significantly higher than that in the HCC group. CD4^+^ was primarily expressed in the TILs cell membrane. No indication of CD4^+^ expression was found in the liver tissue of the control group. The expression of CD4^+^ in TILs was observed in the HCC group and the CIAA group, and the expression of CD4^+^ in the CIAA group was significantly higher than that in the HCC group. IL-10 was mainly expressed in the cytoplasm of HCC cells. There was no obvious expression of IL-10 in the control group, and the expression of IL-10 in the HCC group was significantly higher than that in the CIAA group. PD-1 was mainly expressed in the membrane of TILs. No expression of PD-1 was found in the liver tissue of the control group. In the HCC group, the high expression of PD-1 was observed in HCC tissues. The expression of PD-1 in the CIAA group was significantly lower than that in the HCC group. CTLA-4 was also mainly expressed in the membrane of TILs. No expression of CTLA-4 was found in the liver tissue of the control group. In the HCC group, the high expression of CTLA-4 was observed in HCC tissues. The expression of CTLA-4 in the CIAA group was significantly lower than that in the HCC group (Figure 4). mRNA expression of IL-10 in the HCC group was significantly higher than that in the control group (*P* < 0.0005) and the CIAA group (*P* < 0.0005). No significant difference in the expression of IL-10 was found between the CIAA group and the control group (*P* > 0.05). Moreover, the expression of PD-1 in the HCC group was higher than that in the control group (*P* < 0.05) and the CIAA group (*P* < 0.05). The expression of CTLA-4 in the HCC group was higher than that in the control group (*P* < 0.05) and the CIAA group (*P* < 0.05) (Figure 4).

## 4. Discussion

As an important mechanism of tumorigenesis, immune escape often manifests a decreased level of T lymphocytes in the whole body or in the microenvironment [23]. The capacity of escaping immunosurveillance is of critical importance for the survival of most tumors of solid organs, given that tumors pursue the immune escape mechanism to secure a growth advantage [8]. The improvement of immunosuppression of the body is key to ensure more effective treatment of malignancies. DEN is a carcinogen characterized by its high specificity in inducing HCC, which makes it one of the most commonly used carcinogens at present [24]. In the establishment of the HCC mouse model, the separate use of DEN or CCl_4_ can cause the death of acute disease before the carcinogenesis of HCC. Therefore, DEN and CCl_4_ were used in combination to induce HCC in the study [25, 26].

AFP is a molecular marker of HCC, and its expression in HCC is significantly increased [27]. Our results showed that at mRNA and protein levels, AFP expression was significantly higher in the HCC group than in the control group. Moreover, since roughness and granularity of the surface of liver is an important indicator for the occurrence of the liver cancer, our observation of large masses on the surface of the liver tissue of the HCC group indicates the advanced stages of HCC and the most dismal prognosis of the mice in the HCC group. The histological analysis showed that in the HCC group, the liver cells were disordered, nuclear large, deeply stained, and irregular in shape. Mitotic image and basophilic change of cytoplasm were observed, which was in accordance with the morphological characteristics of HCC. All of these observations jointly confirmed that the effective establishment of the mouse HCC model.

Acupuncture has been applied as a therapeutic option for relieving a variety of maladies in China and other countries. Li et al. reported that the combined treatment of acupuncture and Shenqi Yigan decoction elevated the proportion of CD3^+^, CD4^+^ T lymphocyte subsets, and CD4^+^/CD8^+^ ratio in peripheral blood, whereas it decreased the proportion of CD8^+^, thereby improving the hepatic function of patients with HBV-induced liver fibrosis and reducing the inflammatory reaction [28]. Dong et al. indicated the synergistic effect of acupuncture combined with Chinese herbal medicine, and highlighted evidence-based medical observation for the treatment of primary liver cancer (PLC) [29]. Ling et al. elaborated the guidelines of using transcatheter arterial chemoembolization for advanced PLC in combination with TCM drugs, moxibustion therapy and acupuncture [30]. The clinical efficacy of catgut implantation at acupoints (AICC), a subtype of acupuncture, has also been reported in the treatment of allergic rhinitis (AR), simple obesity, and stroke [31, 32]. Liu et al. reported that CIAA at Dazhui (GV 14), Guanyuan (CV 4), and Zhongwan (CV 12) notably alleviated the neural impairment symptoms and relieved limb spasticity by elevating GABAB expression and reducing mGluR1 levels in the brain stem of rats after stroke [33]. In the current study, we applied CIAA therapy in an animal model of HCC to evaluate its potential efficacy and to explore the underlying mechanism.

Our data show that acupuncture has the effects of regulating immunity, inhibiting the expression of the oncogene, and inhibiting the proliferation of the tumor cells during the treatment. CIAA is the extension and development of acupuncture and moxibustion treatment. Through the continuous stimulation of acupoints, the effect of acupuncture treatment can be amplified [34].

The embedded catgut, as an allogenic substance, can effectively improve the immune function of the human body, yet the effects of CIAA on hepatocarcinogenesis have not been well examined in the HCC model induced by the combination of DEN, CCl_4_, and ethanol. Our results showed that CIAA could decrease AFP protein expression in HCC mice and alleviate the hepatic pathological lesions. These results sufficiently demonstrate the antitumor effect of CIAA treatment on the DEN\CCl_4_\ethanol-induced HCC mice. Subsequently, we further investigated the underlying mechanism of CIAA in attenuating HCC.

Immune escape of tumor cells is one of the main mechanisms of tumorigenesis. Under the action of tumor cells, CD4 cells will produce specific immune tolerance [35]. Then, some of the factors in the microenvironment of the tumor can affect the activity of CTL cells and reduce the sensitivity of CTL to the tumor cells [36]. The negative regulation of IL-10 on immune response in the tumor microenvironment serves to promote the immune escape of tumor cells. In the tumor microenvironment, IL-10 functions to thwart immune responses and promote the immune escape of tumor cells. The research shows that the PD-L1 expression of DCs is upregulated after IL-10 treatment, which may be the mechanism of immune tolerance in the tumor microenvironment [29]. Therefore, we evaluated the aberrantly expression of CD4^+^, CD8^+^, and IL-10 levels. Our results showed that the level of CD4^+^ and CD8^+^ in the CIAA group was increased remarkably in comparison with the HCC group. By contrast, the level of IL-10 in the CIAA group decreased notably compared with that of the HCC group. These data suggested that CIAA might counteract the enhanced level of IL-10, thereby activating the immune function of T lymphocytes in the body.

Programmed death-1 (PD-1) is mainly expressed on the surface of T lymphocytes, B lymphocytes, and macrophages. Programmed death-1 ligand 1 (PD-L1) is expressed on a variety of tumor cells. PD-1 and PD-L1 jointly constitute the immune checkpoint pathway, via which the expression of tumor-infiltrating T lymphocytes is thwarted [37].

CTLA-4 is another important immune checkpoint, which is expressed on the surface of activated T lymphocytes and binds to B7. B7 is on the surface of antigen-presenting cell APC, and it plays the role of immunosuppression [38, 39]. Our studies indicated that the expression of PD-1 and CTLA-4 decreased after CIAA intervention, suggesting that CIAA treatment can improve the local immunosuppression status of HCC by suppressing the expression of PD-1 and CTLA-4. CTLA-4 exhibits stronger affinity when it competitively binds to the B7 molecule against CD28, triggering the inhibitory signal and blocking the activation of T cells by CD28 [14]. In this study, we observed that the expression of PD-1 and CTLA-4 in CIAA-treated HCC mice was decreased, suggesting that CIAA may downregulate the expression of PD-1 and CTLA-4 and restore the inhibition status of the immune checkpoint mediators.

Furthermore, previous research suggested that the activation of the AKT pathway in DEN-induced HCC mice needs about 30 weeks of induction duration [40]. In the HCC model with the activated AKT pathway, there was seriously undermined function of CD4 and CD8 cells [41]. The depletion of CD8 cells and the inhibition of the emergence of an immune cell population aggravate HCC progression. In this study, our western blotting assay shows that the AKT pathway is activated in the DEN\CCl_4_\ethanol-induced HCC mice. Compared with the control group, the expression of CD4 and CD8 in HCC mice indicated that the local natural immunity of the tumor was activated, whereas the increased PD-1 and CTLA-4 expression indicated the occurrence of immune-suppression. The levels of CD4^+^ and CD8^+^ in the CIAA group were obviously increased, in comparison with that of the HCC group. In contrast, the levels of PD-1 and CTLA-4 in the CIAA group were decreased considerably compared with the HCC group. These data suggested that CIAA treatment might play the\ role of restoring the HCC local immunosuppression state, in which the inhibition of the AKT pathway might be implicated.

## 5. Conclusion

CIAA therapy exhibits an antitumor effect on DEN\CCl_4_\ethanol-induced HCC mice, and the mechanism may be attributed to the improvement of local immune status and suppression of the AKT pathway in HCC. Moreover, CIAA downregulates the expression of AFP and reverses the pathological changes of HCC. Therefore, CIAA may be a promising therapeutic method for HCC. More in depth studies should be carried out to clarify and investigate the treatment of tumors with CIAA in the future.

## Figures and Tables

**Table 1:** The evaluation standard of mice condition.

Index/score	1	2	3	4
Body odor	Odor-free	Mild odor	Medium odor	Severe odor
Mental status	Stable	Irritable	Fatigue	Somnolence
Chill and fever	Normal	Cowered	Chill	Arched back and trembling
Respiration	Normal	Panting	Tachypnea	Faint
Fur	Gloss	Matted	Fluffy and erect	Brown and erect
Appetite	Normal	Reduced to 50%	Reduced to 25%	Not at all

**Table 2:** Effect of CIAA treatment on the mortality of HCC mice.

Group	Number of mice (*n*)	Number of death (*n*)	Mortality (%)
Control	10	0	0
HCC	15	6	40
CIAA	15	4	27

## Data Availability

Restrictions apply to the availability of the data, which were used under license for the current study. The data are however available from the authors upon reasonable request.

## Abbreviations

HCC: Hepatocellular carcinoma
CIAA: Catgut implantation at acupoint
DEN: Diethylnitrosamine
CCl4: Carbon tetrachloride
ST36: Zusanli
RN4: Guan Yuan
TIL: Tumor-infiltrating lymphocyte
CTL: Cytotoxic T lymphocyte
DC: Dendritic cells
IL-10: Interleukin 10
CTLA-4: Cytotoxic T lymphocyte-associated antigen-4
PD-1: Programmed death-1
PD-L1: Programmed death-1 ligand 1
AFP: Alpha-fetoprotein.
